# Tribological and Comparative Studies of Ti_3_P + Ti_2_Ni Duplex Layers with TiN Nitride Layers on a Metal Substrate Deposited by PVD

**DOI:** 10.3390/ma19194086

**Published:** 2026-09-24

**Authors:** Michał Sobociński, Marcin Nabrdalik, Paweł Pietrusiewicz

**Affiliations:** 1 Department of Technology and Automation, Faculty of Mechanical Engineering, Czestochowa University of Technology, 42-201 Czestochowa, Poland; marcin.nabrdalik@pcz.pl; 2 Department of Physics, Faculty of Production Engineering and Materials Technology, Czestochowa University of Technology, 42-201 Czestochowa, Poland; pawel.pietrusiewicz@pcz.pl

**Keywords:** hip endoprosthesis, tribology, Ti6Al4V, PVD coatings, TiN, duplex coatings, wear mechanisms, surface engineering

## Abstract

This study assessed whether surface engineering can improve the durability of metal components in hip joint endoprostheses by reducing friction, abrasive wear, and corrosion at the head–socket interface. Single-layer TiN coatings and multilayer TiN + (Ti,N)_3_P + Ti_3_P + (Ti,Ni) coatings were deposited on Ti6Al4V alloy using plasma- and PVD-based processes and tested in combination with Chirulen 1020 ultra-high-molecular-weight polyethylene. Tribological performance, surface condition, microstructure, and chemical composition were evaluated before and after wear testing. The results showed that coating type and deposition method influenced the behavior of the friction pair. The most promising systems were Ti6Al4V coated with titanium nitride by sputtering or plasma spraying and Ti6Al4V with the multilayer coating, both paired with Chirulen 1020. The multilayer-coated Ti6Al4V head combined with a Chirulen 1020 cup provided the most favorable friction-pair performance, indicating its potential to reduce wear-product formation and extend implant service life.

## 1. Introduction

Total hip arthroplasty is one of the most commonly performed orthopedic procedures, aimed at restoring joint function and improving patients’ quality of life. A critical factor determining the long-term performance of hip implants is their durability, particularly the resistance to tribological wear occurring at the articulating interface between the metallic femoral head and the polymeric acetabular cup. Wear processes in this system lead to the generation of debris particles, which may induce inflammatory reactions, osteolysis, and ultimately implant loosening, necessitating revision surgery [1].

Current solutions used in hip replacement surgery do not fully eliminate tribological problems such as friction and wear, which may affect the long-term performance of implants. Therefore, surface modifications aimed at improving the tribological properties of implant components may contribute to reducing wear and potentially extending their service life [1,2].

Under in vivo conditions, the degradation of implant materials is governed primarily by abrasive wear mechanisms, often intensified by corrosive interactions within the physiological environment. The condition of the surface layer of metallic components plays a crucial role in this context. Surface topography parameters, including roughness and waviness, directly influence the nature of tribological contact. Surface irregularities on the femoral head can promote micro-cutting of the polyethylene cup, increasing friction and accelerating material degradation [2]. Consequently, these processes negatively affect both the mechanical stability and longevity of the implant.

To address these challenges, current research focuses on advanced surface engineering techniques aimed at modifying the functional properties of implant materials. Among these, coatings deposited using Physical Vapour Deposition (PVD) methods—such as titanium nitride (TiN) coatings and multilayer or duplex systems (e.g., Ti_3_P + Ti_2_Ni)—have attracted significant attention due to their high hardness, good adhesion, and favorable tribological performance [3,4].

Ti_3_P + Ti_2_Ni coatings are much less common than classic PVD coatings such as TiN, TiAlN, or CrN. In the literature, it appears mainly as a diffusion-reaction composite layer on titanium alloys, particularly biomedical ones (Ti6Al4V), rather than as a typical industrial PVD coating for cutting tools. The layer consists of: a Ti_3_P phosphide phase, a Ti_2_Ni intermetallic phase, and a titanium substrate (most commonly Ti6Al4V). It is typically formed through diffusion phosphiding, thermochemical treatment, or duplex processes (surface modification + an additional bioactive coating). In implants, the Ti_3_P + Ti_2_Ni layer serves to increase corrosion resistance, improve biocompatibility, and act as a transition layer for hydroxyapatite [5,6]. The typical microstructural composition consists of a Ti6Al4V substrate, a Ti_2_Ni diffusion zone, a Ti_3_P layer, and, optionally, hydroxyapatite (HA). The thicknesses of these layers typically range from a few to several dozen micrometers. The formation of such layers ensures high hardness, good chemical resistance, and low biological reactivity.

However, the effectiveness of such coatings depends not only on their chemical composition but also on their microstructure and integrity. In the case of TiN coatings deposited by arc-PVD, surface defects such as pinholes and macrodroplets are commonly observed and may act as initiation sites for corrosion and localized wear [7]. Similarly, duplex coatings containing phases such as Ti_3_P exhibit complex microstructures, and their actual performance requires detailed experimental verification. Additionally, discrepancies between surface-sensitive Energy-Dispersive X-ray Spectroscopy (EDS) and bulk-sensitive X-ray Fluorescence Spectrometry (XRF) analytical techniques highlight the importance of multi-scale characterization in evaluating coating continuity and thickness.

In this study, an attempt is made to evaluate the potential for improving the durability of metallic components in hip joint endoprostheses through the application of advanced surface coatings on Ti6Al4V alloy substrates. The research focuses on the tribological performance of various material pairings, including monolayer TiN coatings and multilayer systems of the type TiN + (Ti,N)_3_P + Ti_3_P + (Ti,Ni), under conditions simulating real implant operation. The aim of this study is to determine the effect of applied surface modifications on the tribological properties of hip joint implant components and to identify material solutions that may contribute to improving the durability of endoprostheses. This study presents an analysis and original research on the factors contributing to increased friction in the contact zone between the femoral head and acetabulum, which revealed that the primary damaging factor in this case is abrasive wear. The corrosive effects of the human body’s internal environment were also considered as a damaging factor [8,9].

## 2. Materials and Experimental Procedure

### 2.1. Materials

The materials used in this study were selected based on their relevance to the tribological performance of hip joint prostheses and included metallic and polymeric components. Particular attention was given to the bearing material used as the counter-specimen in the tribological tests.

Chirulen 1020 is the trade name for medical-grade UHMWPE (Ultra-High Molecular Weight Polyethylene), manufactured by Mitsubishi Chemical Advanced Materials (formerly Quadrant EPP, Lenzburg, Switzerland). It is one of the most important and widely used biomedical materials in the world, used in the manufacture of friction components in joint prostheses (including hip and knee joints) [10,11].

### 2.2. Methods for Preparation of the Samples and Characterization

Experimental investigations were conducted using a “ring-on-block” tribological test and a hip joint simulator, enabling the assessment of wear, friction coefficient, and the characteristics of generated wear debris. Complementary analyses included detailed surface characterization using 3D profilometry and scanning electron microscopy (SEM, JEOL Ltd., Tokyo, Japan) combined with energy-dispersive spectroscopy (EDS) and X-ray fluorescence (XRF, Vanta C, Evident Corporation, Tokyo, Japan) [12]. These techniques enabled the identification of relationships between coating morphology, chemical composition, and tribological behavior, as well as the dominant wear mechanisms occurring in the studied systems.

A Taylor Hobson Form Talysurf Series 50 stationary profilometer (Taylor Hobson Ltd., Leicester, UK) was used to analyze surface roughness, waviness, and shape using 3D measurement software (TalyMap Platinum software, version 5.1.1.5374, Taylor Hobson Ltd., Leicester, UK) [13].

Based on an analysis of the current state of knowledge regarding modern surface engineering methods, a two-layer composite coating—Ti_3_P + Ti_2_Ni—was selected as the material solution to significantly reduce wear.

This coating was deposited using the duplex PVD (Arc-PVD/cathodic arc evaporation) method onto the surface of a Ti6Al4V titanium alloy, which is used for femoral heads, in the form of a Ti_3_P + (Ti-Ni) layer. In addition, studies were conducted on this bilayer coating, which, according to the literature, is characterized by high hardness, good adhesion to the metal substrate, and good tribological properties. For comparison, studies were also conducted on single-layer TiN coatings deposited on the same substrate using the PVD method. A comparison was made between Ti_3_P + Ti_2_Ni duplex layers and single TiN nitride layers obtained by plasma and glow discharge methods and subjected to the Arc-PVD and Nano-Coating on a Ti6Al4V titanium alloy substrate. The designation cathodic arc evaporation refers to the nanostructured PVD coating series of devices, which are used to coat large-sized tools and endoprosthesis components using IET (ion cleaning and etching) technology combined with Arc Evaporation. The designation Nano-Coating (ZY-613) refers to a PVD coating system employing cathodic arc evaporation (Arc-PVD) for the deposition of superhard nanostructured coatings. PVD method was performed with the following process parameters: chamber pressure 10^−3^ mbar, substrate temperature 400 °C, evaporation source—electron beam, reactive gas N2, deposition rate 5 µm/h and VPS method, with the following process parameters: temperature 150 °C, working pressure 10^−3^ mbar, reactive gas N_2_. The following arc-PVD power settings were used: arc current: 100 A per cathode, at a voltage of 25 V and a power of 2.5 kW. Surface preparation prior to depositing the layers consisted of: chemical degreasing, followed by ultrasonic cleaning, vacuum drying, and etching at a polarization voltage of 500 V [14].

Anodizing conditions: 15% H_2_SO_4_ solution, 10 V, 45 min. A 16 nm thick layer with a hardness of 320 HV was obtained. A gold-colored layer was obtained. A so-called barrier layer was formed. It is a compact, dense, and amorphous structure that tightly seals off the substrate from the external environment. The microhardness of the coatings was determined using a Vickers diamond indenter with a 136° face angle, in accordance with ISO 6507-1 [15], using a load of 0.1 kgf (HV0.1). A flowchart of the procedure for sample preparation and coating deposition is shown in Figure 1.

The tribological properties of the obtained coatings, expressed in terms of volumetric wear, were evaluated using a T-05 tribotester (Łukasiewicz Research Network–Institute for Sustainable Technologies, Radom, Poland) based on the roller-block method. Surface roughness and topography were also analyzed for the tested metal rings and femoral heads of the hip joint endoprostheses.

Samples of Ti6Al4V titanium alloy coated with Ti_3_P and Ti_2_Ni were tested in Ringer’s solution, which was used as a physiological test medium [16].

The chemical composition of the tested samples was analyzed using a Vanta^TM^ handheld XRF analyzer (Evident Corporation, Tokyo, Japan). Microstructural observations were performed using a JEOL 6610 LV scanning electron microscope (JEOL Ltd., Tokyo, Japan) at an accelerating voltage of 20 kV and magnifications of ×100, ×5000, and ×10,000. The EDS system integrated with the scanning electron microscope was used to determine the elemental composition and thickness of the deposited coatings [17].

The oxygen (O) and nitrogen (N) contents were determined using an ELTRA ELEMENTRAC ONH-p analyzer (ELTRA GmbH, Haan, Germany) equipped with NDIR and thermal conductivity detectors.

In addition to the Ti6Al4V-based samples, a TiN-coated femoral head with a CoCrMo (ASTM F75 [18]) substrate was included as a reference case in the surface analysis presented in Section 3.4.1. This reference material enabled comparison of the TiN coating continuity and surface defect density between CoCrMo and Ti6Al4V substrates.

The sulfur (S) and carbon (C) contents were determined using an ELTRA ELEMENTRAC CS-i analyzer based on the induction furnace combustion method [19].

#### 2.2.1. Samples Used for Testing on the T-05 Simulator

Table 1 presents a summary of the friction tests performed on the T-05 tester. The T-05 tribotester is a laboratory device for evaluating the tribological properties of materials under controlled sliding conditions.

Figure 2 shows a schematic diagram of the friction node in the T-05 simulator.

The tests on the T-05 tribotester were conducted under lubricated sliding contact with forward motion using a “ring-block” configuration, with the operating parameters presented in Table 2. The general testing methodology and the block-on-ring configuration were based on ASTM G77-17(2022) [20], while the specific test parameters were selected according to the investigated material combinations and lubricated operating conditions. Similar tribological investigations using the T-05 tribotester in a block-on-ring configuration have also been reported by Kubik et al. [21].

Most material pairs were tested over 182.000 cycles, which corresponds to 20.008 km of friction distance. After 22.750 cycles, corresponding to 2.501 km, each sample (pad) and counter-sample (ring) was weighed to determine the amount of wear on the friction materials.

During system operation, the friction force, lubricant temperature, and sample (pad) temperature were measured. The wear particles present in the lubricant reservoir were drained after the completion of the 20.008 km cycle and examined under a transmitted-light microscope connected to a digital image analyzer.

#### 2.2.2. Samples Tested on a Hip Joint Simulator

Figure 3 illustrates the shapes of acetabulum cup and head used for testing on the hip simulator. Figure 4 shows a diagram of the hip joint simulator.

The hip joint simulator tests were conducted with the following parameters:
-type of friction pairendoprosthesis ‘head—cup’-static load1200 N-dynamic load2300 N-relative sliding speed0.132 m/min-endoprosthesis stem length130 mm-lubricating fluidRinger’s solution.

The wear products generated at the “prosthesis head–acetabular cup” contact point were washed away by the lubricating fluid and transported along with the fluid through a special channel into a container. After draining the fluid, it was possible to analyze the quantity and size of the particles and observe them under a transmitted-light microscope.

## 3. Results and Discussion

### 3.1. Results of the T-05 Tribological Tests

Figure 5 shows a summary of the average volume loss of the test specimen and the counter-specimen (n = 5), where the standard deviation is ±0.0036.

Figure 6 shows the mean maximum and mean minimum values of the coefficients of friction recorded during the tests for the material combinations under assessment (n = 5), where the standard deviation is ±0.04.

Furthermore, based on the sample size (n = 5) and the calculated standard deviation (SD = 0.04), the 95% confidence intervals (CI) for the mean friction coefficients were determined using Student’s t-distribution (d_f_ = 4, t = 2.776). The resulting margin of error for the mean values is ± 0.050. Additionally, the 95% confidence interval for the standard deviation itself was evaluated using the Chi-square distribution, yielding a range of [0.024; 0.115].

Similarly, for the wear volume loss data (DV), the standard deviation was calculated as SD = 0.0036 cm^3^ (n = 5). The corresponding 95% confidence interval (CI) for the mean wear values was evaluated using Student’s t-distribution (df = 4), establishing a margin of error of ±0.0045 cm^3^. The 95% confidence interval for the wear standard deviation itself ranges from 0.0022 to 0.0103 cm3 based on the Chi-square distribution.

The coatings obtained through processes Arc-PVD and Nano-Coating had thicknesses of approximately 2 µm and hardnesses of 3200 HV and 4300 HV, respectively. Process Arc-PVD produced a rough coating due to the formation of microdroplets on the surface.

During the separation session, magnetic filters were activated, which deflected the trajectory of the ions striking the workpiece, while heavy, inert droplets settled on the filter walls. This resulted in the physical separation of the pure plasma from macroscopic contaminants. The analyzed regions exhibited compositions compatible with Ti_3_P- and Ti_2_Ni-type phases; however, their crystallographic identity could not be conclusively established by the methods employed in this study.

The pair exhibiting a high coefficient of friction throughout the entire test cycle was the combination of Chirulen 1020 + Ti6Al4V (anodically oxidized at 10 V for 45 min) and Chirulen 1020 + Ti6Al4V (without surface treatment). The coefficient of friction for this pair ranged from μ = 0.0875 to 0.0625, while for the pair with an anodized titanium ring, it ranged from μ = 0.125 to 0.1. During testing of the Chirulen 1020 + anodically oxidized Ti6Al4V material combination, a large amount of polyethylene wear particles was generated. These particles became lodged between the mating surfaces, causing an increase in the coefficient of friction.

Titanium particles from the Chirulen 1020 + Ti6Al4V composite, which had not undergone surface treatment, played a similar role. The large amount of wear debris from the titanium ring and the relatively large amount of polyethylene particles resulted in a high coefficient of friction. Tests of both of these pairs were terminated after a friction distance of S = 10 km due to seizure of the friction surfaces. Distinct surface scratches were visible on the titanium rings. The Chirulen 1020 + Ti6Al4V pair, subjected to processes Arc-PVD and Nano-Coating, aimed at depositing the following layers: TiN + (Ti,N)_3_P + Ti_3_P + (Ti,Ni), achieved surprisingly good friction and wear performance. This multilayer ring, which interacted with a polyethylene known as Chirulen 1020, exhibited only slight wear. The volume loss of the ring at the end of the test cycle was ΔV = 0.0001883 [cm^3^]. The coefficient of friction for this pair ranged from μ = 0.0225 to 0.065. For comparison, the coefficient of friction for the Chirulen 1020 + CoCrMo material combination, which has been widely used in arthroplasty to date, was μ = 0.0625 to 0.025. A similar coefficient of friction was achieved by Chirulen 1020 + Ti6Al4V pairs coated with sputter-deposited titanium nitride (TiN), for which the coefficient of friction ranged from μ = 0.03 to 0. 0625, and Chirulen 1020 + Ti6Al4V coated with plasma-deposited titanium nitride, for which the coefficient of friction was μ = 0.056 to 0.075. The above graphs indicate that the most promising pairs for the future are Ti6Al4V coated with sputtered or plasma-deposited titanium nitride in combination with Chirulen 1020, and the Ti6Al4V titanium alloy coated with a multilayer TiN + (Ti,N)_3_P + Ti_3_P + (Ti,Ni) in combination with Chirulen 1020.

The results obtained are highly consistent with previous research on implant materials, as cited in the literature referenced in the article. This confirmed that the test was correctly calibrated [4,10,22].

It was observed that titanium surfaces became worn after approximately 10 km, leading to the generation of so-called ‘third-body wear’. A thin oxide layer (16 nm) did not allow for the long-term maintenance of wear resistance. Breaching this barrier quickly led to a drastic increase in friction, which is consistent with the fracture mechanics of thin layers on a soft substrate. Trapping macro-droplets from the plasma eliminates the roughness that damages the polymer. The hardness achieved for the multi-layer coating exceeds that of standard TiN coatings and explains the excellent results, which are comparable to those of CoCrMo alloy.

### 3.2. Results Obtained Using the Hip Joint Simulator

The friction coefficients for different friction pairs obtained on the hip joint simulator are illustrated in Figure 7.

Tests on the simulator take into account the complex kinematics of gait (movements in multiple planes) and the specific spherical spatial geometry (head-acetabulum), which provides a picture much closer to actual clinical conditions within the human body [1,10,23].

The minimum and maximum values of the friction coefficients for the tested friction pairs are illustrated in Figure 8.

In tests conducted on a hip joint simulator for the material combinations presented, friction coefficients were obtained that were similar to those of the tested CoCrMo + Chirulen 1020 pair currently used for endoprosthesis components. The coefficient of friction for the “reference” pair ranged from μ = 0.3 to 0.25. Compared to this material pair, the other combinations achieved similar values. The lowest friction coefficient values were achieved by the pair consisting of a Chirulen 1020 polyethylene cup and a Ti6Al4V stem coated with multilayer TiN + (Ti,N)_3_P + Ti_3_P + (Ti,Ni) coatings. The coefficient of friction for this pair was μ = 0.222 to 0.218. A Chirulen 1020 bearing mating with a Ti6Al4V alloy head coated with plasma-deposited titanium nitride achieved equally good results. The coefficient of friction for this pair was μ = 0.252 to 0.205. All the pairs tested, having completed the running-in phase (approx. 300.000–400.000 cycles), show satisfactory stabilization of their operating parameters. The standard number of cycles is 5 million (in accordance with ISO 14242-1) [24]. For research purposes, when testing new coatings (to closely observe the running-in phase), shorter tests are carried out, e.g., up to 1 million cycles. The data suggests that the higher coefficient of friction (μ) corresponds to the start of the test, whilst the lower value represents the stabilized value. Table 3 presents a quantification of the coefficients of friction obtained for the friction pairs under investigation.

### 3.3. Analysis of Surface Condition of Metallic Components

To further assess the effects of tribological interaction, the surface condition, roughness, and topography of the tested metallic components were analyzed. Measurements were performed for the metal ring used in the T-05 tribotester and for the metal femoral heads tested against polyethylene components. The analysis included both three-dimensional surface topography and two-dimensional profile parameters.

Sample results of this analysis are presented graphically in Figure 9 and Figure 10. Figure 9 shows an example of the surface topography of a metal ring interacting with a polyethylene sample after testing on the T-05 tribotester, along with roughness parameters. Figure 10 shows an example of the surface topography of a metal endoprosthesis head interacting with a polyethylene cup, along with the corresponding roughness parameters. In order to assess surface roughness, five 3D surface measurements were taken at different locations so that the mean (R_a_, S_a_) and standard deviation (SD) would accurately describe the surface condition. To compare the implants before and after the tribological tests, and to assess changes in the parameters, data were collected from five samples (heads/rings) tested under the same conditions. When analyzing the surface of a sample component, individual geometric defects can be identified. These are distortions that arise during tribological interaction as a result of surface wear. Such a deviation (approx. 11 μm) drastically distorts certain profile parameters. It can therefore be concluded that, under the operating conditions of this system, this could lead to accelerated wear of the interacting components. R_sk_ = −0.3 indicates that the profile (surface) distributes loads well and wears more slowly, while the valleys act as reservoirs for lubricant. The R_ku_ = 6.74 value indicates a so-called leptokurtic distribution, which means that the profile is relatively flat, but there are very sharp, steep peaks or deep troughs here and there, as confirmed by the presence of a single high peak in the 2D profile.

The analysis was divided into a spatial (3D) assessment in accordance with ISO 25178 [25] and a profile (2D) assessment in accordance with ISO 4287 [26]. A distinct peak (elevation) exceeding 10 μm indicates contamination or adhered wear debris (so-called material transfer). S_a_ = 1.99 μm and S_q_ = 2.47 μm—that is, the mean and standard deviation of surface height—indicate that the surface, on a macro scale (including its cylindrical shape), exhibits noticeable waviness/curvature. S_sk_ = 0.430, meaning that the skewness (asymmetry of the height distribution) is positive, which indicates a slight predominance of sharp peaks over valleys.

Figure 10 shows that this sample has a much smoother surface than the ring sample.

Visually, the surface appears very uniform and smooth, with no distinct deep scratches, which is crucial for medical implants designed to minimize friction. A sharp peak approximately 1.5 μm high and a smaller peak of 0.4 μm represent microscopic contaminants on the mirror-like surface of the head (e.g., a speck of dust during measurement) or a local defect/inclusion. By analyzing the surface roughness parameters of the metal disc sample and comparing them to those of the metal head of the endoprosthesis, it can be concluded that the extent of tribological wear in the latter case is significantly lower. Key roughness parameters such as R_z_ and R_a_ differ drastically in both cases, favoring the metal endoprosthesis head. Based on the analyses of the metal samples, it can be concluded that the surface condition parameters are consistent with those typically observed in this type of application. For example, the roughness parameters of the ring used for testing on the T-05 tribotester changed from R_a_ = 0.509, R_z_ = 4.14 (before the test) to R_a_ = 0.796, R_z_ = 4.81 (after the test). For the endoprosthesis heads tested on the hip joint simulator, the parameters changed from R_a_ = 0.02, R_z_ = 0.51 (before the test) to R_a_ = 0.19, R_z_ = 1.35 (after the test).

These findings demonstrate that both bilayer number and deposition time critically determine the mechanical and functional response of Ti/TiN multilayers. The results provide practical guidelines for the reliable characterization and design of multilayer coatings for biomedical and aerospace applications. The obtained results are consistent with previous studies on the tribological behaviour of biomedical alloys and TiN-coated components. In the present study, the roughness of the femoral heads increased from R_a_ = 0.02 and R_z_ = 0.51 to R_a_ = 0.19 and R_z_ = 1.35, whereas the ring specimens showed a greater increase from R_a_ = 0.509 and R_z_ = 4.14 to R_a_ = 0.796 and R_z_ = 4.81. Zivic et al. [2] demonstrated that the friction and wear of Ti6Al4V strongly depend on its microstructure, while Basgul et al. [3] reported very low surface roughness of retrieved TiN-coated femoral heads (S_a_ = 37.3 nm) and scratches of 0.3–1.3 µm depth. The surface defects observed in the present study are also comparable with PVD-related defects reported by Łapaj et al. [22], including pinholes, droplets and local delamination. Furthermore, Liu et al. [4] and AbuAlia et al. [7] confirmed that TiN deposition technology significantly affects coating properties and can improve the wear resistance of Ti6Al4V and CoCrMo alloys. Thus, the present results confirm the importance of surface condition and coating technology in controlling the tribological performance of biomedical components.

### 3.4. XRF, EDS and Scanning Microscopy Analysis

The mechanical strength of the mating surfaces of joint prostheses is a key parameter that determines how long the implant can function in the body. In this area, SEM, EDS, and XRF methods make it possible to identify signs of wear and material degradation on the surfaces being examined.

#### 3.4.1. Coating Morphology and Composition—TiN-Coated/CoCrMo Head

The study examined hip prosthesis heads made of a CoCrMo alloy coated with a layer of TiN.

Figure 11 presents representative SEM images of the TiN-coated CoCrMo head, while Table 4 summarizes the corresponding EDS-derived elemental composition of the coating surface and substrate. Together, these results allow direct correlation between the observed surface morphology and its local chemical composition, as discussed below.

Table 5 shows the elemental composition of the TiN coating surface determined by X-ray fluorescence.

As shown in Figure 11a (×5000), the surface is predominantly smooth and uniform with a fine columnar-granular structure typical of TiN deposited by arc-PVD. However, occasional defects in the form of pinholes and macrodroplets with diameters of 2–5 µm are observed.

An SEM image at 10.000× magnification (Figure 11b) shows the sample surface with a uniform and compact morphology. The analyzed surface is characterized by a fine-grained structure, with no clearly visible cracks, pores, or surface defects on the micrometer scale.

The topographic contrast is low, suggesting a high degree of microstructural homogeneity and the absence of local variations in height. The observed surface texture indicates a uniform distribution of grains smaller than 1 µm, which may indicate stable conditions during the material/coating fabrication process.

Consistent with these morphological observations, EDS analysis (Table 2) performed on smooth surface areas revealed a composition very close to stoichiometric TiN (Ti:N = 75.64: 23.76 wt.%; theoretically 77.4: 22.6%). The substrate signal (Co, Cr, Mo) is practically absent, indicating that in defect-free areas the coating is continuous and sufficiently thick to completely shield the substrate.

In contrast to EDS, XRF analysis (larger measurement volume) showed only 23.93% Ti (Table 3). This difference stems from the presence of defects (pinholes and macrodroplets) visible in the SEM micrographs—XRF radiation penetrates through these voids and primarily detects the composition of the CoCrMo ASTM F75 alloy.

XRF chemical composition analysis revealed a relatively low titanium content (23.93% by weight), even though a TiN coating is present. Given the significantly greater penetration depth of XRF radiation compared to EDS (approx. 10–50 µm for XRF vs. 1–5 µm for EDS), this result indicates an effective coating thickness of 2–4 µm or a significant proportion of surface defects, through which the signal originates mainly from the CoCrMo substrate [27,28].

The observed microdefects and the discrepancy between the EDS and XRF results are fully consistent with the literature on TiN coatings deposited by arc-PVD on orthopedic implants [22,23]. Similar pinholes and macrodroplets have been reported in all previous studies of TiN-coated femoral heads and are considered an inherent feature of this technology. These defects may serve as potential sites for corrosion initiation, coating delamination, or accelerated polyethylene wear under in vivo conditions.

#### 3.4.2. Coating Morphology and Composition—Process Cathodic Arc Evaporation Duplex Head

Figure 12 shows SEM micrographs of the surface of a TiN coating deposited on the head of a hip prosthesis.

An analysis of Figure 12 reveals that, unlike the TiN/CoCrMo head described above, this surface is heavily defective—numerous irregular pits, deep cavities, grooves, and cracks ranging in size from 1 to 10 µm are visible, and the coating structure is porous and non-uniform.

According to the specification, the Process Arc-PVD Duplex tip has a duplex structure: Ti_3_P + (Ti-Ni) transition layers and a TiN coating [29,30]. This is confirmed by the EDS results in Table 4: the presence of 13.37 wt.% P on the surface (EDS) unequivocally confirms the presence of the Ti_3_P phase (Table 6). The phosphorus content is consistent with a partially exposed or very thin Ti_3_P layer (theoretical composition of Ti_3_P: ~82% Ti, ~18% P; the measurement result indicates a mixture of Ti_3_P + Ti-6Al-4V substrate).

Table 6 further shows that nitrogen (N) was detected only locally (18.49% by mass over a large area), indicating a discontinuous outer TiN coating. Nickel (Ni) was not detected in any measurement (Table 6 and Table 7)—the (Ti-Ni) layer is either below the detection limit or absent in the tested sample.

The high density of surface defects (pinholes, grooves, pits) despite the presence of the Ti_3_P layer suggests that the deposition process of the transition layers and TiN did not ensure full continuity and tightness of the coating.

Comparing the XRF results in Table 7 with the EDS data above, when comparing the two heads, the coating on the Process Arc-PVD Duplex head (Ti6Al4V + Ti_3_P) exhibits a significantly higher number and larger size of defects than the coating on the TiN/CoCrMo head (without the Ti_3_P coating). The presence of the Ti_3_P phase was confirmed (13.37% P), but it did not prevent the formation of numerous defects—unlike the more homogeneous coating on the CoCrMo substrate [29,30].

The Ti_3_P-containing coating on the Ti6Al4V femoral head exhibited a higher density and size of surface defects than the TiN coating on the CoCrMo head. Although Ti_3_P was confirmed by the phosphorus content of 13.37%, its presence did not prevent coating discontinuities. This finding is consistent with previous studies reporting pinholes, droplets, blisters, and local delamination in PVD TiN coatings on femoral heads [22]. The increased defect density may be related to the more complex duplex architecture and the Arc-PVD deposition process, in which droplet formation and surface irregularities are recognized limitations [14]. The lower defect density and more homogeneous morphology of the TiN/CoCrMo coating may therefore indicate better coating integrity, which is relevant to its long-term tribological performance [3,22].

#### 3.4.3. Wear-Induced Surface and Compositional Changes—Ti6Al4V Ring

Figure 13 shows SEM images of Ti6Al4V rings with different surface conditions: (a) a worn ring with a duplex coating, (b) a polished Ti6Al4V ring, and (c) a Ti6Al4V ring after test 05. All images were obtained in SEI mode at an accelerating voltage of 20 kV and a magnification of 100×.

A worn Ti6Al4V ring with a duplex coating (Figure 13a) exhibits distinct directional wear marks aligned with the sliding direction, accompanied by localized material removal and associated contaminants. Such surface features are characteristic of titanium alloys subjected to dry sliding conditions and are commonly associated with a combination of abrasive and adhesive wear mechanisms. Repeated sliding promotes the fragmentation of surface layers and the formation of a discontinuous multilayer, a phenomenon that has been observed in Ti6Al4V alloys under comparable contact conditions.

In contrast, the polished Ti6Al4V surface (Figure 13b) is characterized by a smooth and uniform morphology with shallow, parallel polishing marks and no visible signs of degradation, which is typical of Ti6Al4V surfaces that have undergone mechanical treatment prior to tribological testing. This confirms that the surface damage observed in the worn specimens is caused by tribological interaction, not by the initial surface preparation.

The Ti6Al4V ring after tests of T-05 (Figure 13c) reveals continuous and well-developed abrasive grooves, indicating a more stable wear regime dominated by mild abrasive wear. Similar groove-like morphologies have been observed in Ti6Al4V alloys sliding against metal counter-faces under moderate loads, where abrasion is the dominant wear mechanism.

These morphological observations are corroborated by the compositional data in Table 8: EDS analysis of the worn surface with a duplex coating reveals significant differences in composition between the light and dark areas. The light areas are dominated by Ti, Al, and V, which corresponds to the composition of the base alloy, while the dark areas show a clearly increased content of oxygen and carbon, along with detectable amounts of Fe, Mn, and other secondary elements. Elevated oxygen concentrations strongly suggest the occurrence of tribo-oxidation processes, which play a key role in the wear behavior of Ti6Al4V alloys during dry sliding. The presence of iron is most likely associated with material transfer from the bearing surface, a phenomenon frequently observed in tribological systems involving titanium alloys.

XRF measurements (Table 9) confirm that the chemical composition of the Ti6Al4V alloy remains stable for all tested samples, regardless of surface condition. This contrast between the EDS and XRF datasets demonstrates that the compositional changes detected by EDS are limited to the near-surface region and are associated with oxidation and material transfer caused by wear, rather than bulk material modification, which is consistent with the results of previous studies combining surface and bulk analysis methods.

#### 3.4.4. Bulk Chemical Stability—Gas Analysis (O, N, C, S)

In addition to SEM, EDS, and XRF analyses, the tested samples were characterized using gas analysis to determine their oxygen, nitrogen, carbon, and sulfur content (Table 10). The results obtained provide supplementary information on the chemical composition of the Ti6Al4V alloy in the near-surface layer and in the bulk, under various surface conditions.

As shown in Table 10, the oxygen content remains relatively stable for all samples, ranging from 0.205 to 0.23% by weight, indicating that neither polishing nor tribological testing led to a significant increase in the oxygen concentration within the material. This suggests that the oxidation processes observed on worn surfaces using EDS are localized surface phenomena rather than oxidation of the alloy in bulk.

The nitrogen and carbon contents are also comparable across all tested samples, with nitrogen values ranging from approximately 0.012% to 0.014% by weight and carbon content below 0.02% by weight. The absence of a significant increase in nitrogen content confirms that no significant formation of nitrides occurred during the tests, while the low and stable carbon concentration indicates limited carbon uptake from the environment or the material on the opposite surface.

Sulfur is present only in trace amounts in all samples, at levels below 0.003% by weight, confirming that sulfur-related contamination did not affect the observed tribological behavior.

Importantly, after testing of T-05, the Ti6Al4V ring exhibits a gas content comparable to that of the polished reference sample, which further confirms that tribological interaction did not cause any measurable changes in the material’s composition. Gas analysis, together with XRF results, confirms that the compositional changes detected by EDS on worn surfaces are limited to the surface or near-surface regions and are associated with tribo-oxidation and material transfer, rather than with chemical modification of the alloy within its bulk.

## 4. Conclusions

The tribological tests demonstrated that inappropriate surface modification of Ti6Al4V may significantly deteriorate the performance of the Ti6Al4V–Chirulen 1020 friction pair. In particular, anodizing at 10 V for 45 min increased the coefficient of friction from 0.0625–0.0875 for the untreated alloy to 0.10–0.125 and promoted severe polyethylene wear.

The multilayer coatings provided substantially higher hardness (3200–4300 HV) than conventional TiN coatings (typically 2000–2500 HV), while their multilayer architecture improved resistance to crack propagation and coating delamination.

The most promising friction pair identified in the present study was Chirulen 1020 polyethylene combined with a Ti6Al4V femoral head coated with the TiN + (Ti,N)_3_P + Ti_3_P + (Ti,Ni) multilayer system. The results indicate that appropriate surface modification and low surface roughness can substantially reduce wear and support the formation of a stable lubricating film.

The obtained results confirm the potential of multilayer PVD coatings for improving the tribological performance of titanium alloy components for hip endoprostheses. However, the present study is limited by the laboratory character of the tests and the need for further validation under physiological tribocorrosion conditions, including assessment of long-term coating stability and possible ion release, particularly for patients sensitive to nickel.

Further research should focus on optimizing coating architecture and deposition parameters and on verifying the long-term performance of the selected friction pairs under clinically representative loading and lubrication conditions.

## Figures and Tables

**Figure 1:**
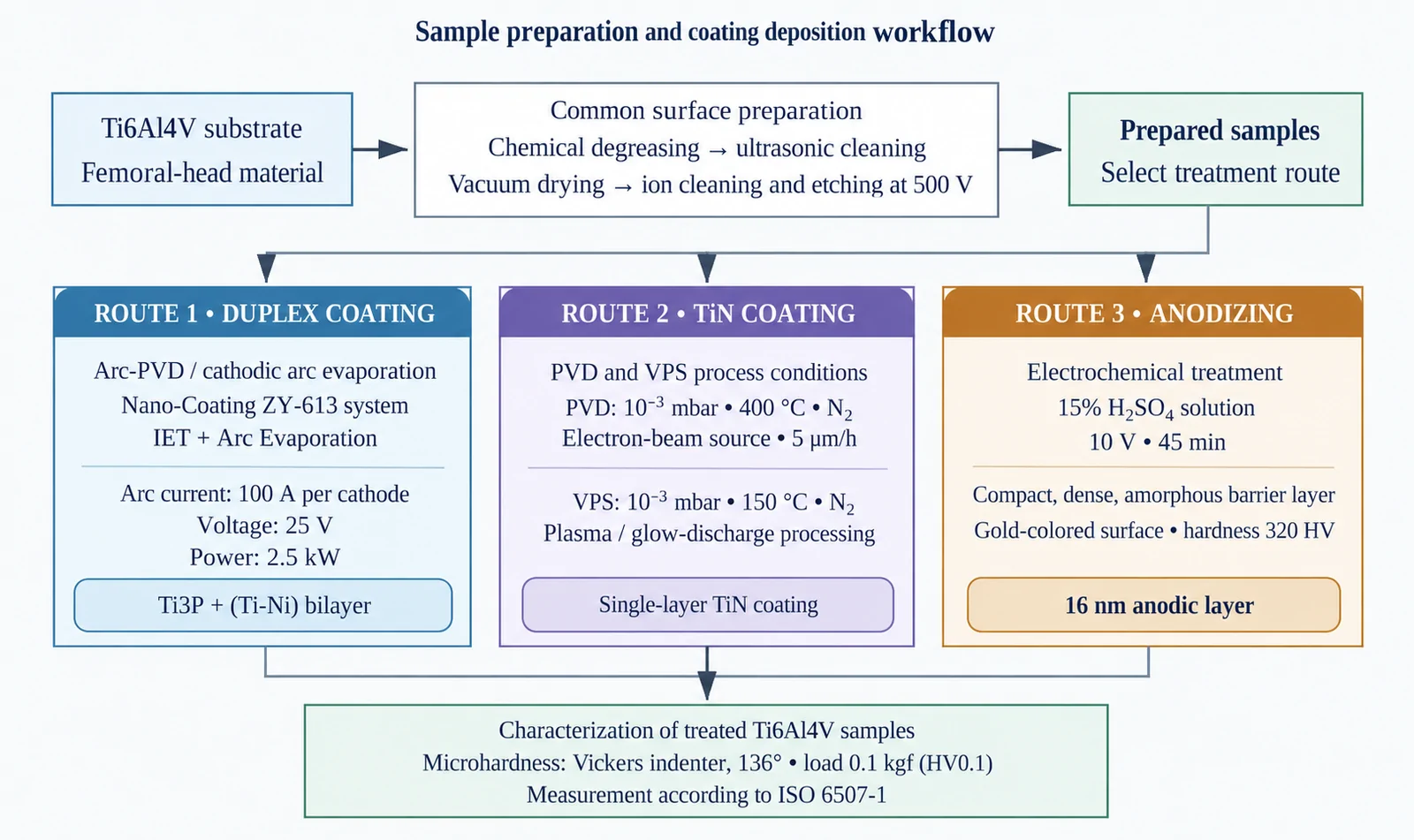
Flowchart of the procedure for sample preparation and coating deposition.

**Figure 2:**
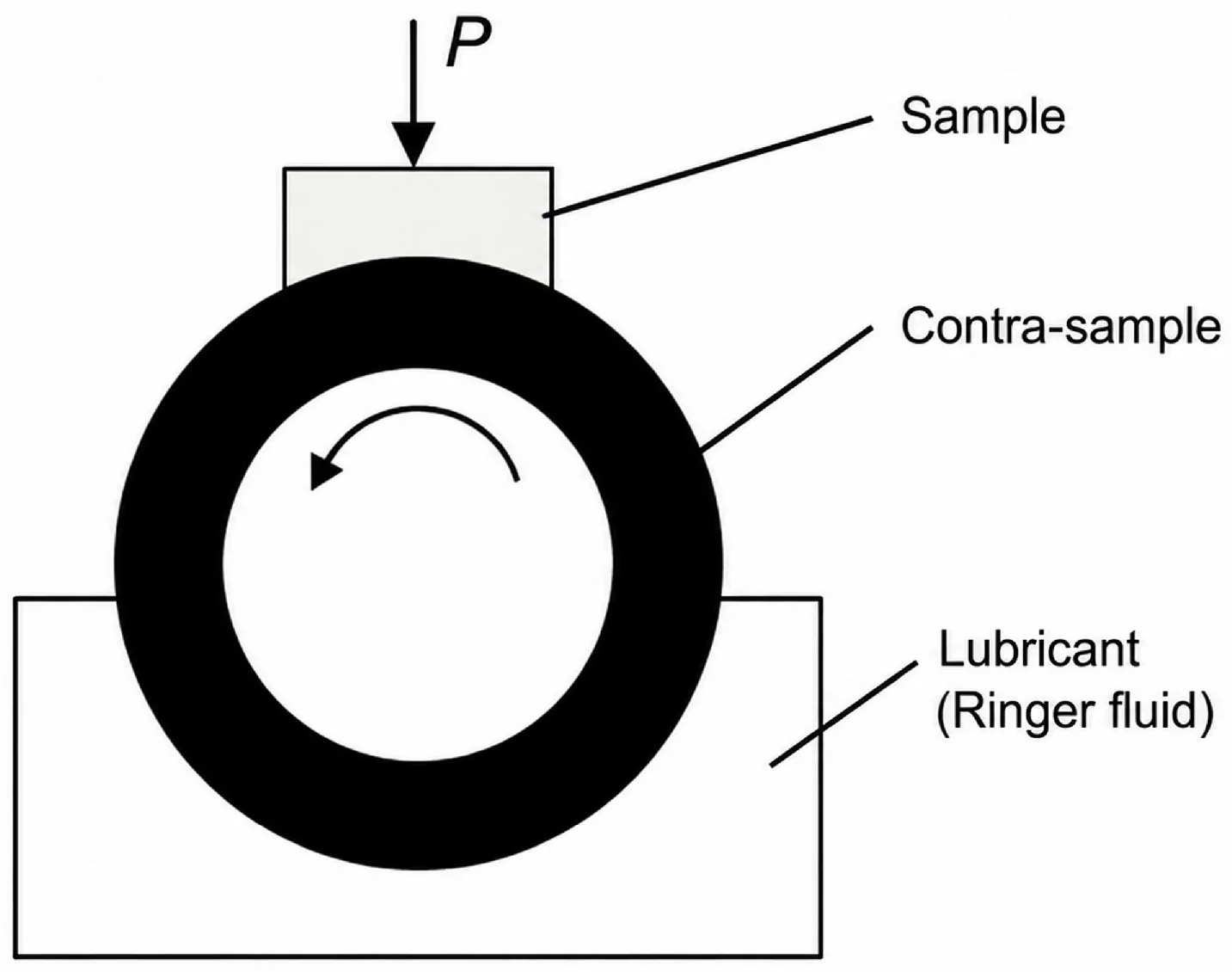
Schematic of the friction node in the T-05 simulator.

**Figure 3:**
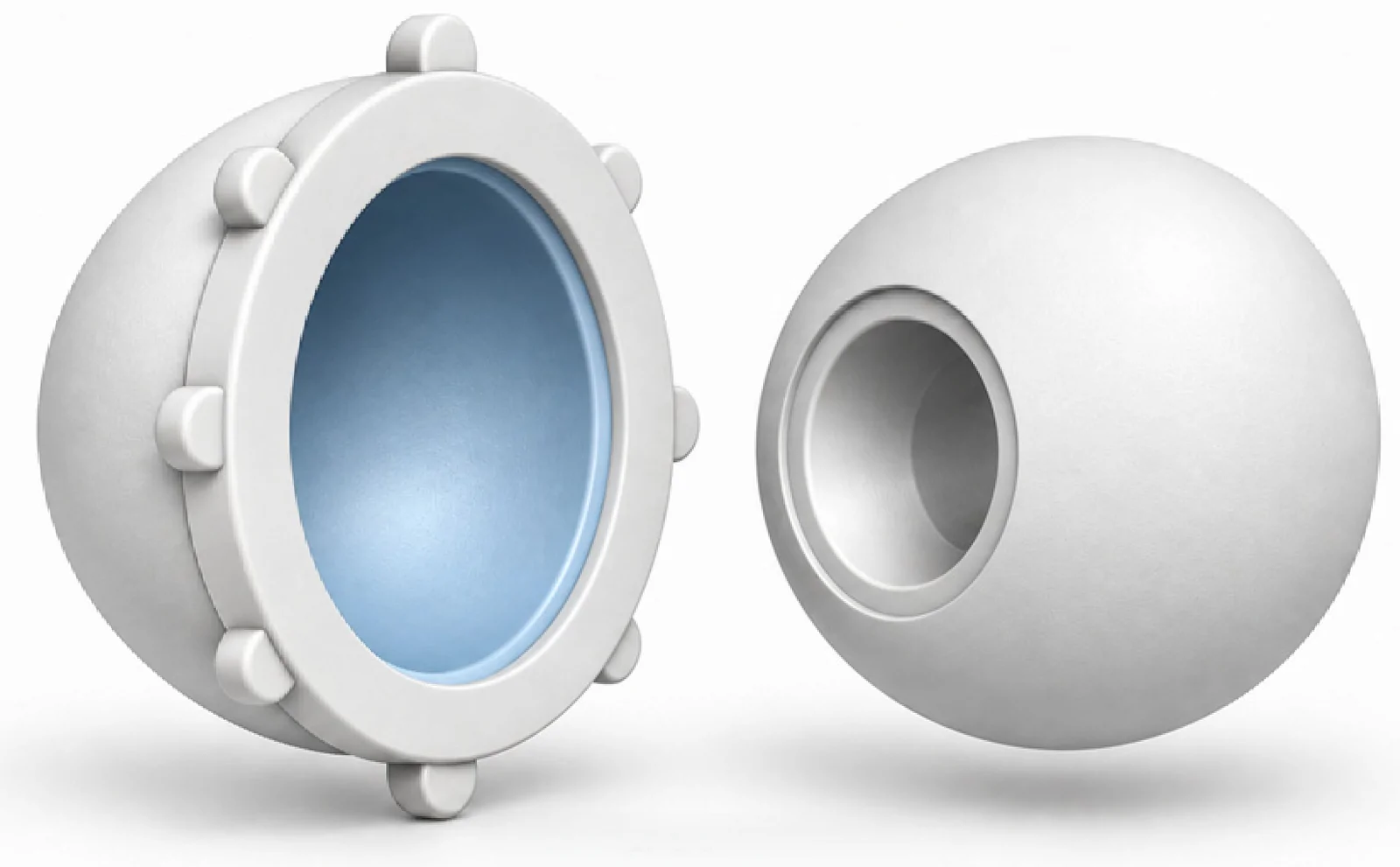
Shapes of the cup and head with a diameter of ø 28 mm and ø 32 mm.

**Figure 4:**
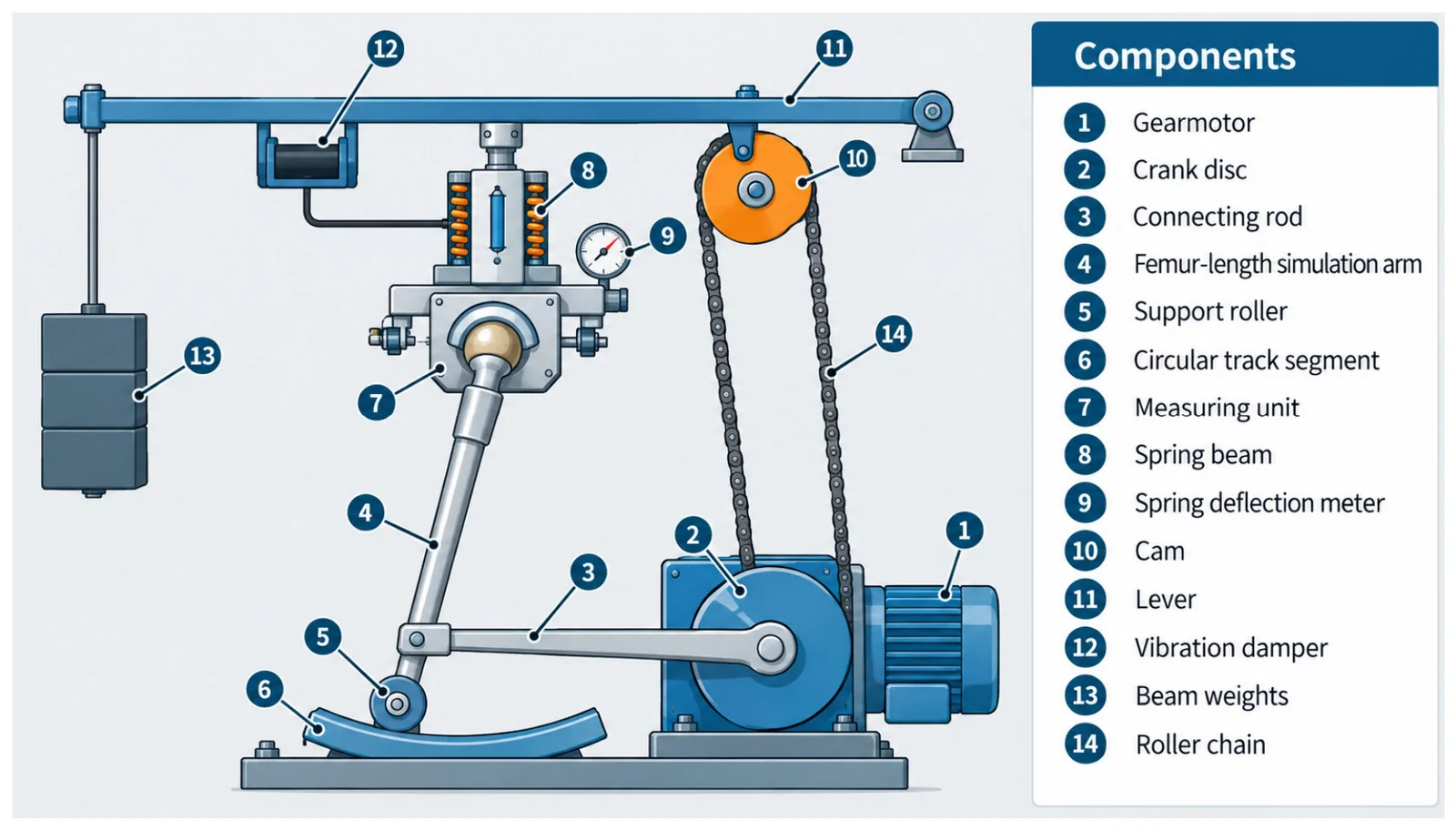
Schematic diagram of the stand for testing durability of human hip joint endoprosthesis.

**Figure 5:**
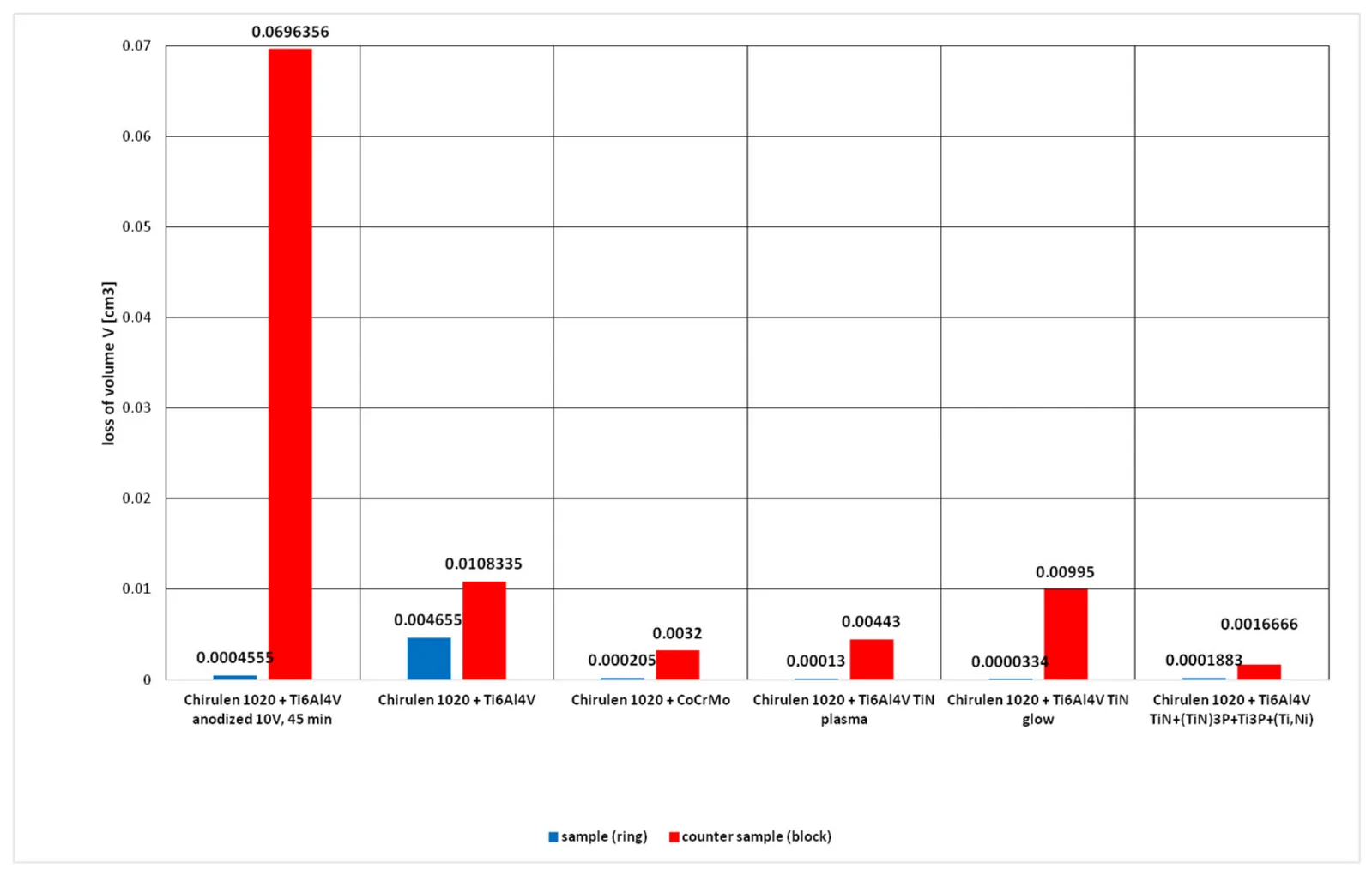
Summary of sample and counter-sample consumption.

**Figure 6:**
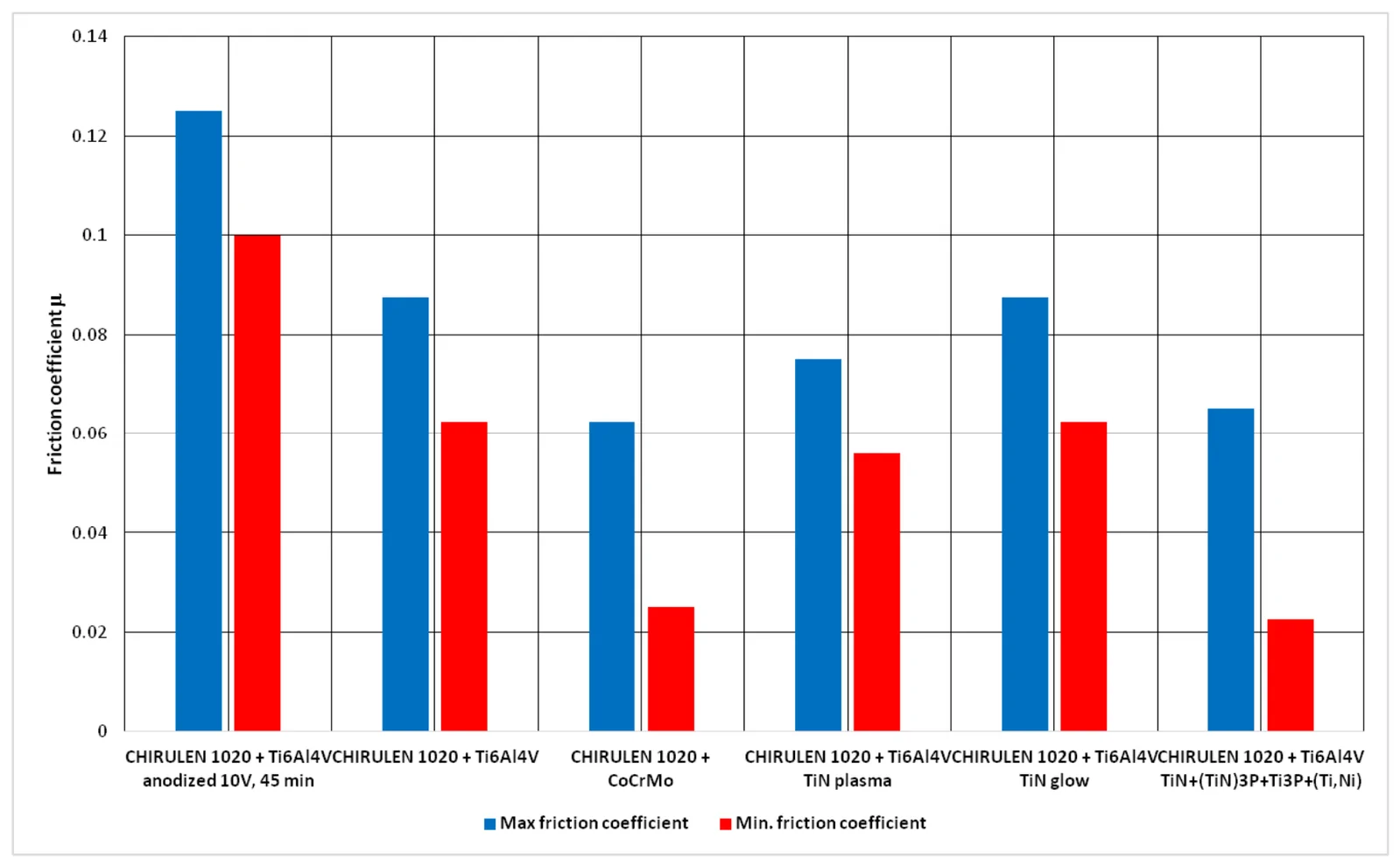
Summary of friction coefficient during testing.

**Figure 7:**
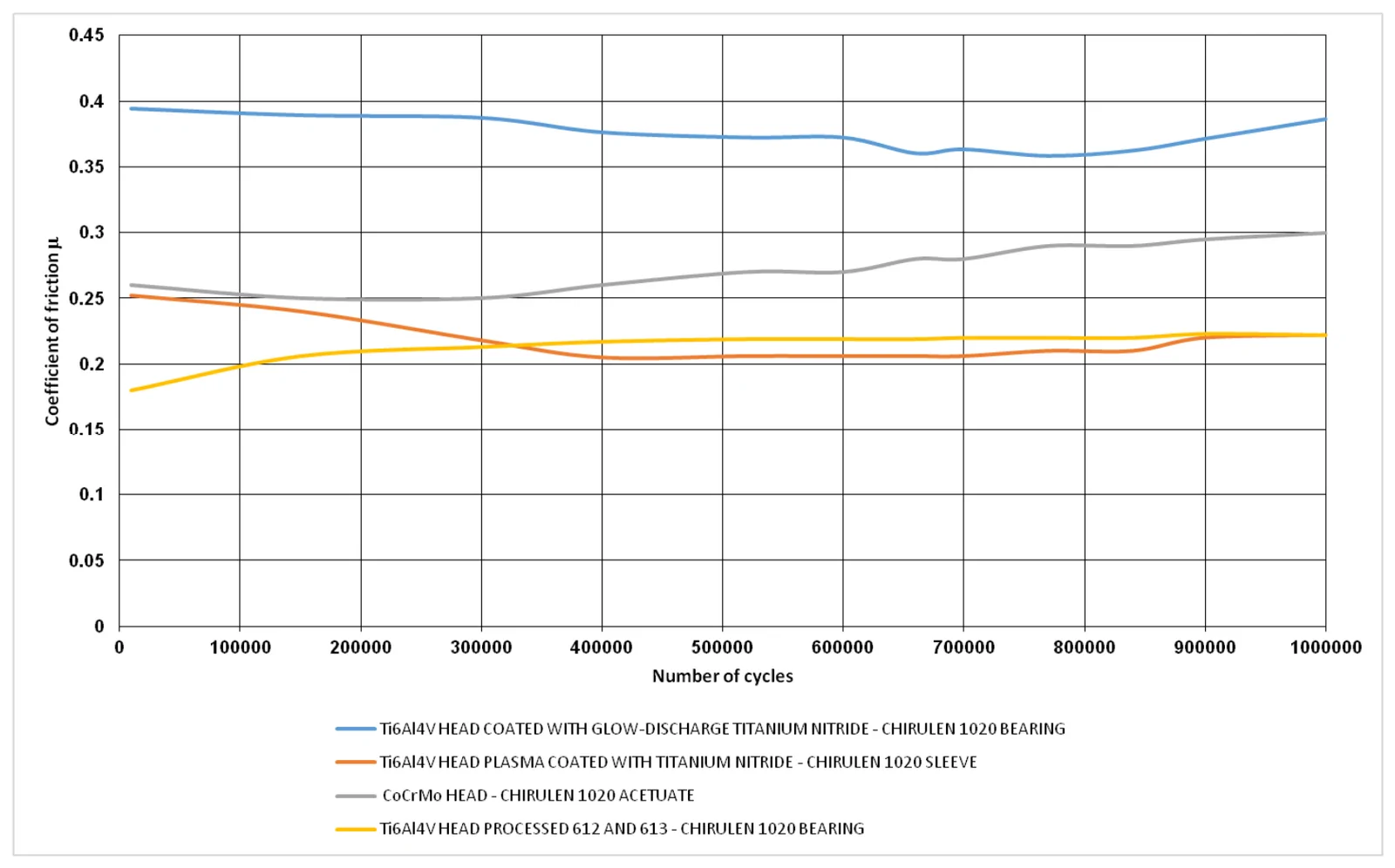
Friction coefficients for various friction pairs obtained on the hip joint simulator.

**Figure 8:**
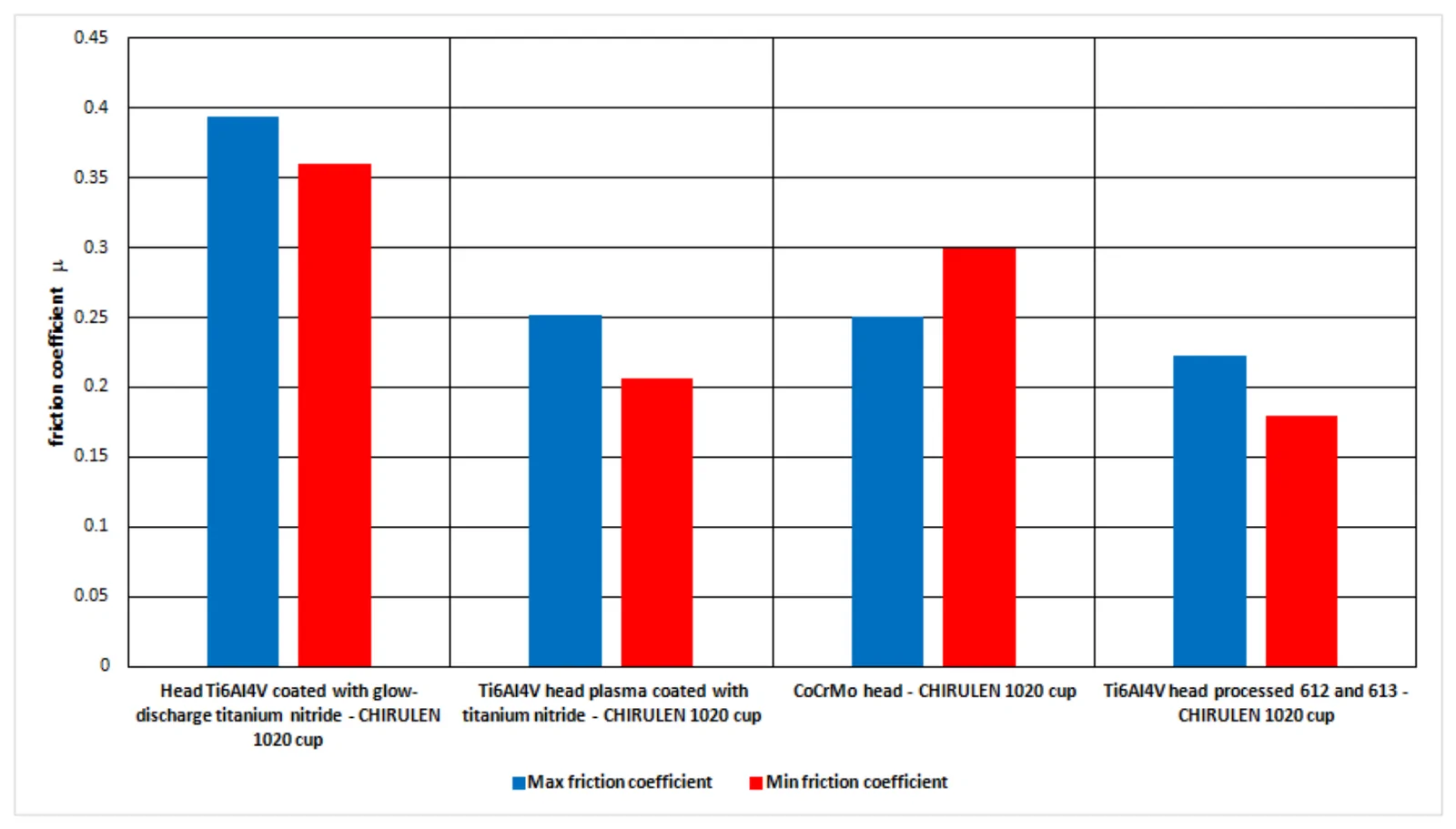
The friction coefficient values for the tested friction pairs.

**Figure 9:**
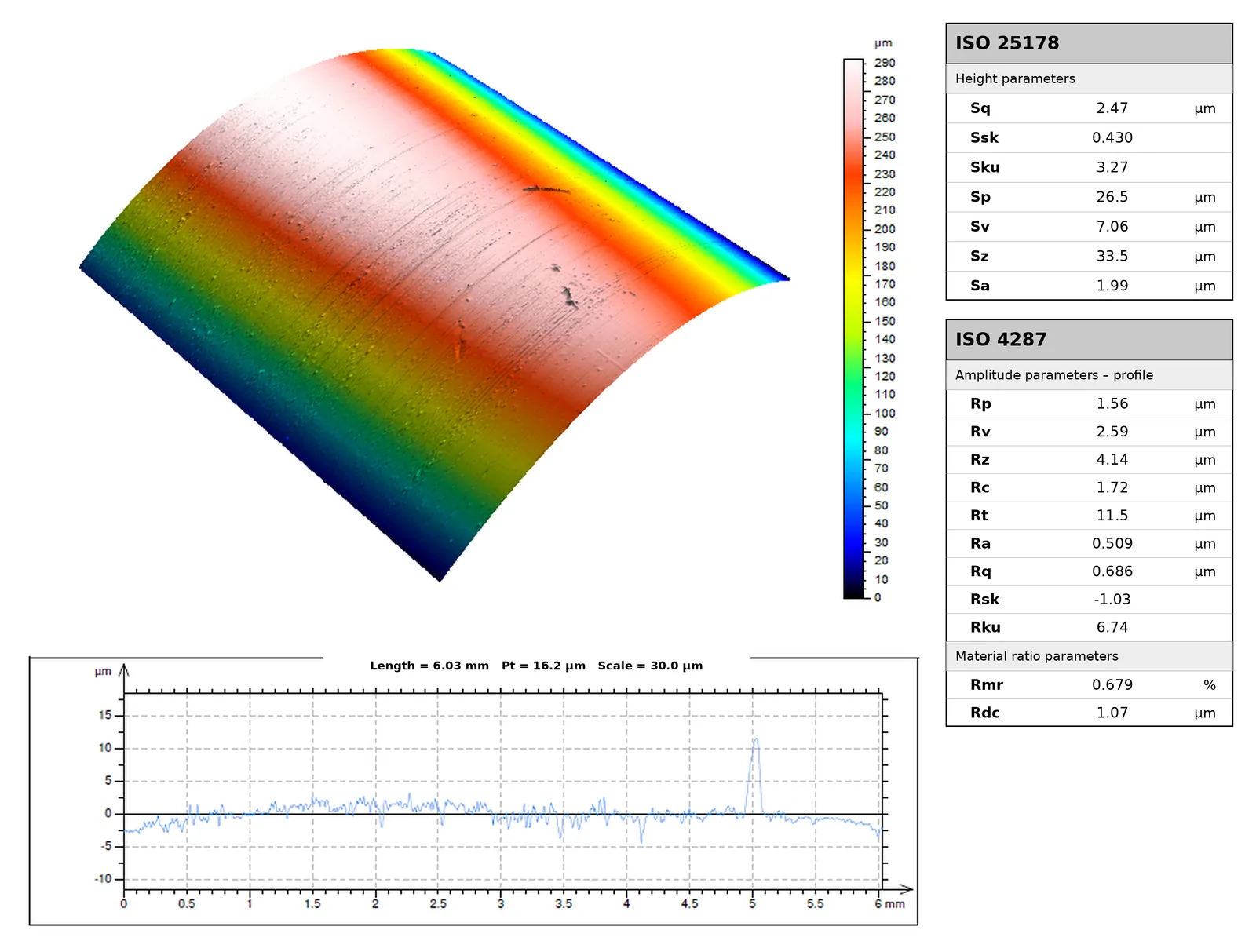
Example analysis of surface condition: roughness, waviness and shape of the sample (ring).

**Figure 10:**
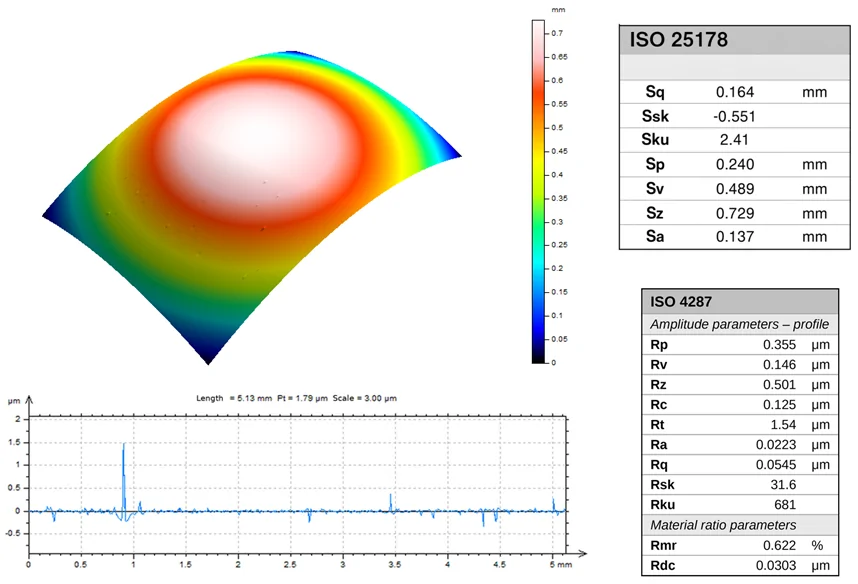
Example analysis of the surface condition: roughness, waviness and shape of the head of a metal endoprosthesis.

**Figure 11:**
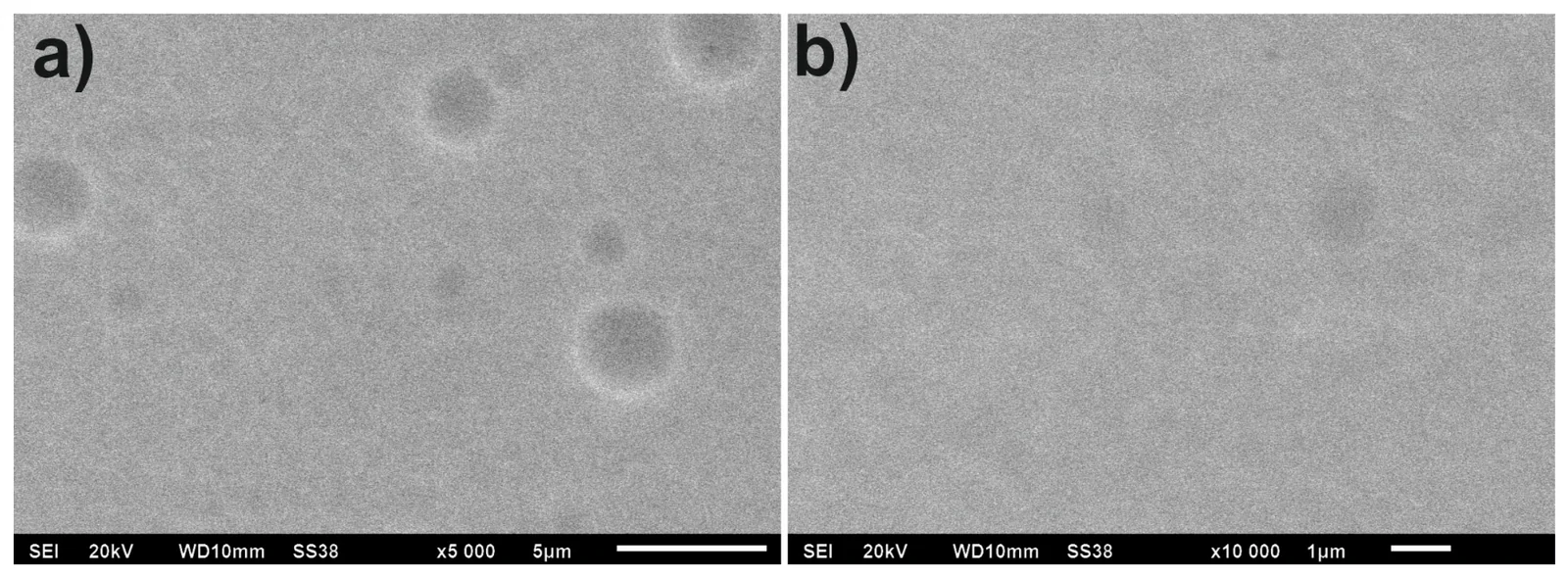
Representative SEM images (SEI, 20 kV) of the surface of a TiN coating deposited on the head of a CoCrMo alloy (ASTM F75) hip endoprosthesis, included as a reference sample representing a widely used clinical TiN/CoCrMo coating–substrate combination, distinct from the Ti6Al4V-based systems that are the main focus of this study. (**a**) Magnification ×5000, scale bar 5 µm. (**b**) Magnification ×10,000, scale bar 1 µm.

**Figure 12:**
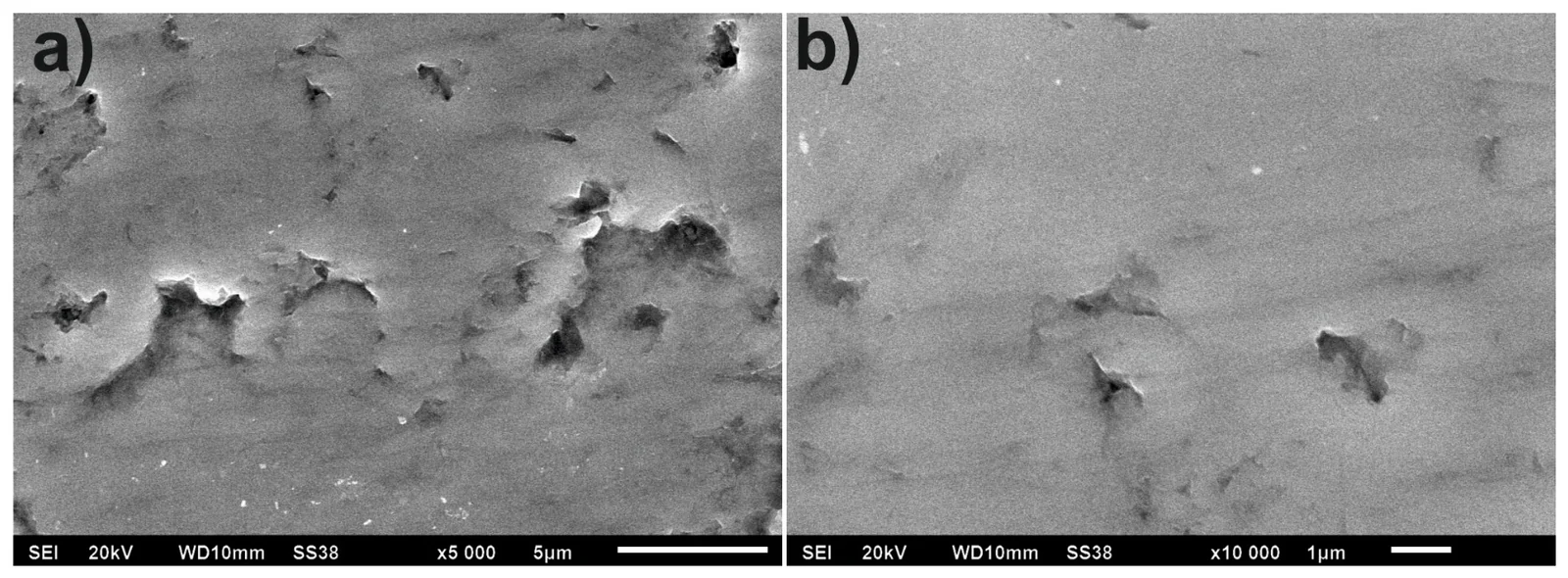
Representative SEM micrographs (SEI, 20 kV) of the surface of a TiN coating deposited on the head of a Process Arc-PVD Duplex hip endoprosthesis (Ti-6Al-4V substrate). (**a**) Magnification ×5000, scale bar 5 µm. (**b**) Magnification ×10,000, scale bar 1 µm.

**Figure 13:**
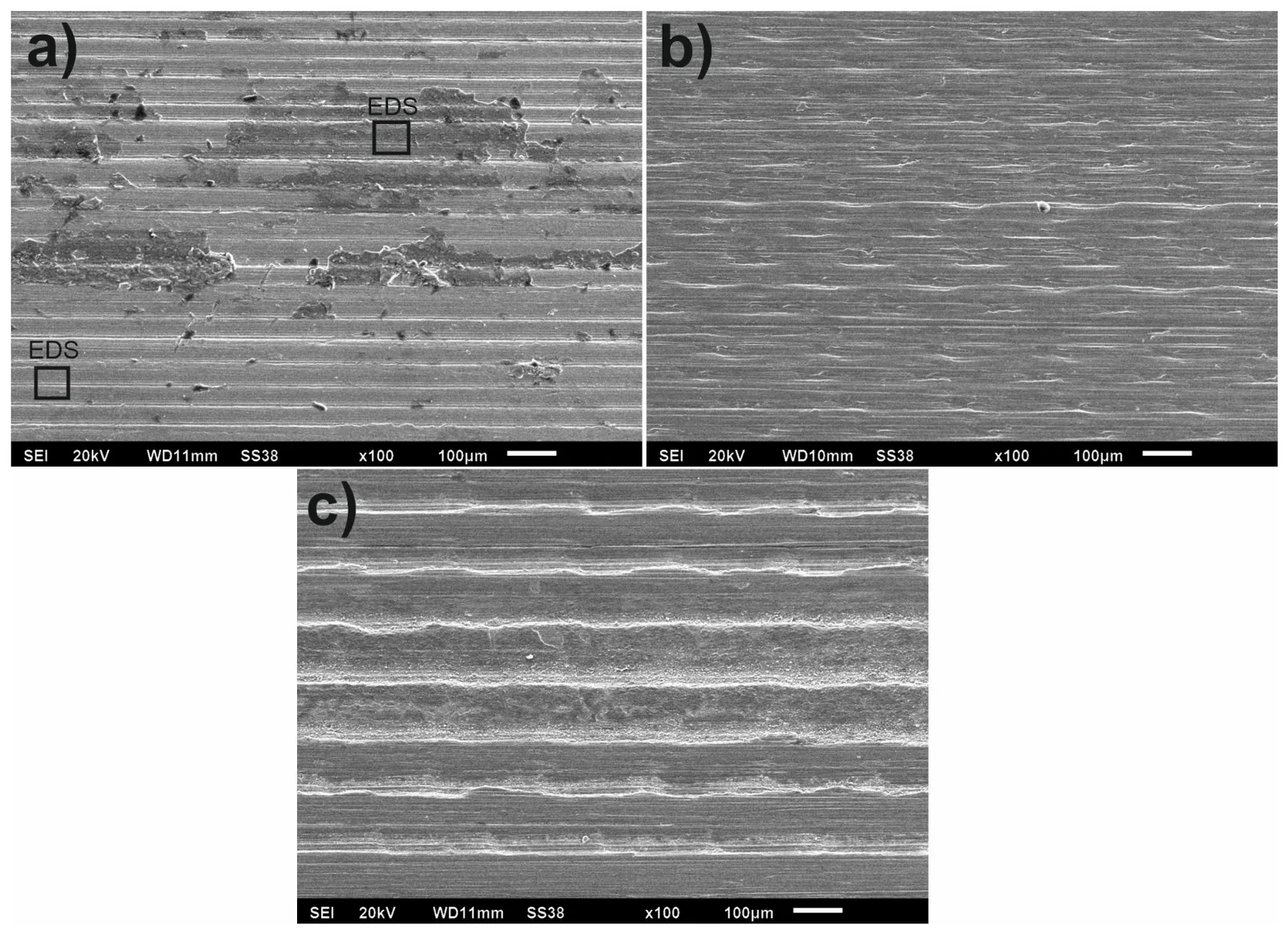
SEM images of Ti6Al4V rings obtained in SEI mode at 20 kV and 100× magnification: (**a**) a worn ring with a duplex coating showing visible wear marks and contaminants; (**b**) a polished Ti6Al4V surface with uniform polishing marks; (**c**) a Ti6Al4V ring after Test 05 with continuous wear grooves.

**Table 1:** Summary of the analyzed friction pairs.

Sample (Ring) Material	Counter-Sample Material (Block)
Ti6Al4V titanium alloy anodically oxidized at 10 V, 45 min Ti6Al4V alloy CoCrMo alloy Ti6Al4V titanium alloy coated with plasma-applied titanium nitride Glow-discharge titanium nitride-coated Ti6Al4V alloy Ti6Al4V alloy subjected to trial Arc-PVD and Nano-Coating TiN + (Ti,N)_3_P + Ti_3_P + (Ti,Ni)	CHIRULEN 1020 [11]

**Table 2:** Summary of the parameters of the tribotester T-05.

Parameter	Description/Value
type of friction pair	ring-block
load on the friction node	600 N and 800 N
stress	6 N/mm^2^ and 8 N/mm^2^
rotational speed	0.91 rpm
number of cycles	22,750 (2.501 km)
permissible resistances	120 N
lubricating liquid	Ringer’s solution

**Table 3:** A comparison of the results obtained from tests on the coefficient of friction between the femoral head and acetabular cup using a hip joint simulator.

Friction Pair (Head—Cup Chirulen 1020)	Max μ (Running-in Phase)	Min μ (Stabilized)	Change in Friction Coefficient	Tribological Evaluation
CoCrMo (current standard/reference)	0.300	0.250	16.6%	reference
Ti6Al4V (TiN—plasma coating)	0.252	0.205	18.6%	very good
Ti6Al4V (multi-layer coating: TiN + …)	0.222	0.218	1.8%	best

**Table 4:** Summary of the analyzed friction pairs.

Results EDS	Ti	N	Co	Cr	Mo	Si
Head TiN	75.64	23.76	0.6	-	-	-
Head TiN cross-section	0.18	-	63.17	29.72	5.96	0.98

**Table 5:** Elemental composition of the TiN coating surface determined by X-ray fluorescence (XRF).

Results XRF	Ti	Co	Cr	Mo	Mn	Fe	Ni	Cu
Head TiN	23.93	46.13	23.27	4.925	0.967	0.157	0.616	-
Head TiN cross-section	-	63.67	28.25	6.008	0.429	0.504	0.452	0.025

**Table 6:** Elemental composition of the TiN coating surface and substrate determined by EDS (SEM, 20 kV).

Results EDS	Ti	N	Si	Al.	P	V
Head Process Arc-PVD Duplex	85.26			1.48	13.37	
Head Process Arc-PVD Duplex large measurement area	78.86	18.49	0.08	0.44	2.14	
Head Process Arc-PVD Duplex cross-section	89.57			6.21		4.14

**Table 7:** Elemental composition of the surface and cross-section determined by XRF.

Results XRF	Ti	Al	V	Cr	Mo	Fe	Ni	Cu	Zr
Head process Arc-PVD duplex	95.53	0.93	3.17		0.005	0.194	0.168	0.006	0.002
Head process Arc-PVD duplex cross-section	90.05	5.12	4.33	0.079	0.004	0.265	0.142	0.008	0.002

**Table 8:** EDS analysis results for the hip joint endoprosthesis cup.

Results EDS	Ti	Al	V	C	O	Fe	Si	S	Ca	Mn	Na	K
Ring Ti6Al4V used (coating duplex) Bright measurement area	90.62	6.15	3.23	-	-	-	-	-	-	-	-	-
Ring Ti6Al4V used (coating duplex) Dark measurement area	28.09	1.12	1.15	4.36	45.01	18.2	0.24	0.35	0.13	0.66	0.22	0.18
Ring Ti6Al4V polished	89.44	6.77	3.8	-	-	-	-	-	-	-	-	-
Ring Ti6Al4V test 05	70.6	5.12	3.1	3.03	15.75	2.4	-	-	-	-	-	-

**Table 9:** XRF analysis results for a hip replacement cup.

Results XRF	Ti	Al	V	Cr	Mo	Fe	Ni	Cu	Zr	Si	Sn	Zn
Ring Ti6Al4V used (coating duplex)	88.72	6.72	3.9	0.077	0.005	0.52	0.009	0.007	0.004	0.028	0.007	0.004
Ring Ti6Al4V polished	88.76	6.61	4.2	0.113	0.005	0.24	0.019	0.005	0.002			
Ring Ti6Al4V test 05	88.82	6.58	4.2		0.005	0.27	0.018	0.009	0.002	0.043		

**Table 10:** The oxygen, nitrogen, carbon, and sulfur content (wt.%) were determined by gas analysis for Ti6Al4V samples with different surface conditions.

Sample	O%	N%	C%	S%
Head Porus Arc-PVD duplex	0.23 (2)	0.06 (1)	0.018 (6)	0.0009 (2)
Ring Ti6Al4V polished	0.21 (1)	0.012 (1)	0.016 (3)	0.0008 (2)
Ring Ti6Al4V used coat duplex	0.225 (2)	0.014 (1)	0.019 (6)	0.0022 (3)
Ring Ti6Al4V T-05	0.205 (3)	0.012 (1)	0.015 (2)	0.0007 (1)

## Data Availability

The original contributions presented in the study are included in the article; further inquiries can be directed to the corresponding author.
